# Does Interventional Pain Management Play a Role in the Treatment of Cervical Post-Surgical Neuropathic Pain?

**DOI:** 10.7759/cureus.48996

**Published:** 2023-11-18

**Authors:** António Paiva, José Bernardo Ferreira, Simão Serrano

**Affiliations:** 1 Physical Medicine and Rehabilitation, Centro de Medicina de Reabilitação da Região Centro - Rovisco Pais, Tocha, PRT; 2 Physical Medicine and Rehabilitation, Centro Hospitalar de Leiria, Leiria, PRT

**Keywords:** ultrasound guided nerve block, nerve hydrodissection, neuropathic pain treatment, schwannoma, superficial cervical plexus block

## Abstract

Post-surgical neuropathic pain is still an underdiagnosed medical condition with persistent pain occurring in 10-50% of patients submitted to surgery. We present a case of a 52-year-old patient with persistent paracervical, supraclavicular, and upper chest neuropathic pain after the excision of a massive deep right cervical tumor, concluded to be an accessory spinal nerve schwannoma. A thorough physical and ultrasound examination helped conclude injury of the superficial cervical plexus. Therefore, an ultrasound-guided hydrodissection of several neuromas was performed at the level of the superficial cervical plexus. After three procedures, pain and quality of life scores improved, with a reduction of anxiety and depression symptoms. Due to the positive response, the patient was referred for a peripheral nerve stimulator implantation, allowing self-control of pain, in a home setting.

## Introduction

Superficial cervical plexus (SCP) originates from the anterior branches of spinal nerves C2 to C4 and gives rise to four terminal branches: auricular magnus, occipital minor, transverse cervical, and suprascapular nerves. SCP is responsible for the sensory innervation of a large anatomical area, including skin and superficial structures of the cervical region, submandibular area, earlobe, supraclavicular area, and upper chest [1,2]. Post-surgical neuropathic pain (PSNP) following lateral cervical surgery and SCP injury has been previously demonstrated [3]. PSNP prevalence may reach 40% of the patients due to the anatomical changes that may occur during surgery or after radiotherapy [4,5].

SCP block provides effective anesthesia and analgesia for the head and neck regions and is often used for loco-regional anesthesia during anterolateral approaches in neck surgeries, including carotid endarterectomies, lymph node dissection, and plastic surgery [6,7]. Ultrasound-guided cervical plexus nerve block technique is preferred over the landmark-based technique and the authors aim to demonstrate that it can also be used for chronic pain treatment purposes, enabling real-time visualization of anatomical structures and needle movement, avoiding a needle insertion that is too deep and the inadvertent puncture of neighboring structures, reducing complication rates [8,9].

## Case presentation

We describe the clinical case of a 52-year-old male, with no relevant pathological or surgical history, who underwent surgical excision of a right paracervical tumor, suspected to be a deep cervical adenopathy. However, histopathology revealed it to be a schwannoma, possibly of the XI cranial nerve (accessory spinal nerve) according to the surgical report. In the post-surgery period, the patient started to develop intense pain in the right supraclavicular region, also affecting the function of the right upper limb. Pharmacological treatment was then initiated with pregabalin 225 mg/day.

One month after surgery, the patient was referred to the Physical and Rehabilitation Medicine (PRM) consultation where he presented with severe and persistent neuropathic pain, refractory to medical treatment. Neuropathic descriptors, according to the Douleur Neuropathique 4 Questions scale, included electric shock, tingling, numbness, and itching. During physical examination, upon inspection, only right suprascapular fossa atrophy was noted, possibly due to trapezius muscle hypotrophy. A right scapular dyskinesis was observed with excessive lateralizing scapular protraction (lateral winging). Regarding shoulder range of motion (ROM), flexion, and lateral abduction were limited to 110º, due to scapular abnormal motion and shoulder pain with no external and internal rotation deficits.

The upper limb motor strength and myotatic reflexes were normal, except for right shoulder elevation with motor strength grade 2 Medical Research Council (MRC) scale. In the sensory exam, the patient presented with brushing/allodynia and touch hypoesthesia on the entire cutaneous territory of the right anterolateral neck, the ante and retro-auricular areas, as well as the skin overlying and immediately inferior to the clavicle on the upper chest wall. Touch and pinprick anesthesia in the earlobe region was noted. No sensory abnormalities were noted in the right upper limb. Circadian rhythm sleep disorder was present due to pain and quality-of-life impairment with anxiety and depression symptoms.

At the pain consultation, the pregabalin daily dose was increased to 300 mg, tapentadol 50 mg (suspended due to adverse effects), and amitriptyline 10 mg. A lidocaine patch of 5% a day was used without symptom relief. Capsaicin 8% topical patch was applied for three consecutive treatments, at eight-week intervals, with mild outcomes in neuropathic descriptors. A multimodal therapy approach including occupational therapy and physical therapy was introduced with progressive rehabilitation of the scapula dyskinesis.

**Figure 1:**
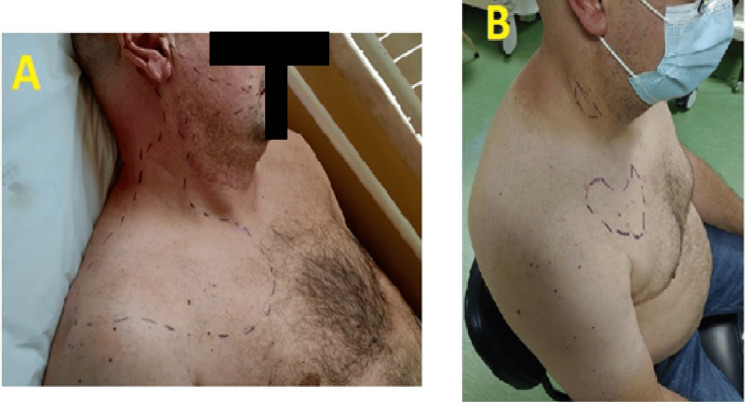
Area of hypoesthesia and allodynia demarcated by the dashed line (A) Before performing the first procedure; (B) Four weeks after the third procedure

Clinical examination suggested the diagnosis of iatrogenic injury of the right accessory spinal nerve and of the right SCP (Figure 1A). Nerve conduction study and needle electromyography revealed a right accessory nerve injury, classified as partial axonotmesis, moderate to severe severity.

**Figure 2:**
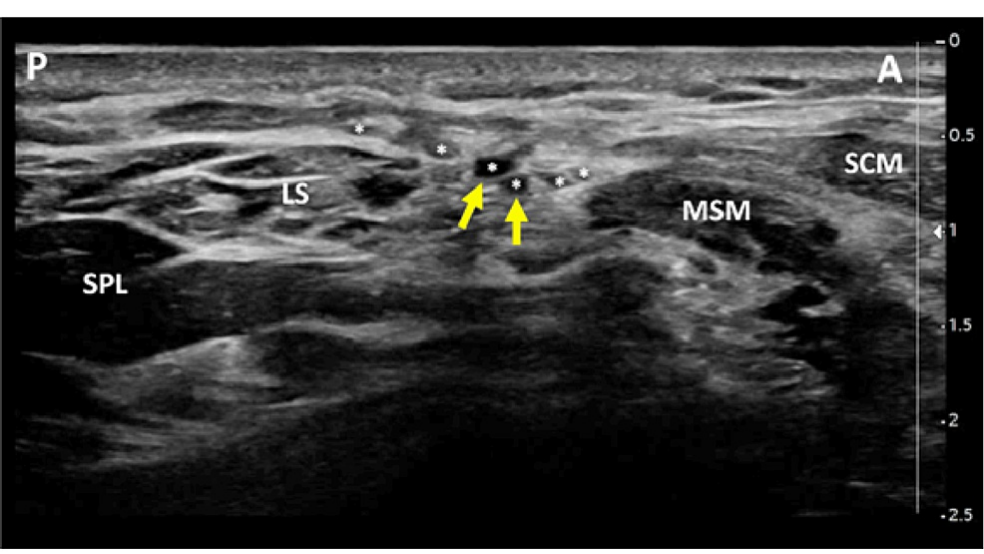
Superficial cervical plexus ultrasound imaging (transverse view) Note the superficial cervical plexus branches (asterisks) with some showing signs of enlargement and possibly neuroma formation (yellow arrows). P: posterior; A: anterior; LS: levator scapulae muscle; SPL: splenius capitis muscle; MSM: middle scalene muscle; SCM: sternocleidomastoid muscle

At the ultrasound examination and SCP evaluation, in close proximity to the surgical scar, several branches of the cervical plexus, lying superficial to the prevertebral fascia and posterior to the sternocleidomastoid muscle, were swollen, showing a hypoechoic enlarged ecostructure, highly suggestive of neuroma formation (Figure 2).

Then, after getting proper signed informed consent, an ultrasound-guided hydrodissection of the right SCP was performed, deep into the sternocleidomastoid muscle. The procedure was performed using 4 mL of local anesthetic (ropivacaine 2%), 7 mL of saline solution (dextrose 5%), and 1 mL of corticoid (methylprednisolone 40 mg/ml), in a total volume of approximately 12 mL (Video 1).

**Video 1:** Superficial cervical plexus ultrasound-guided hydrodissection LS: levator scapulae muscle; SPL: splenius capitis muscle; MSM: middle scalene muscle; SCM: sternocleidomastoid muscle

After the first procedure, the patient reported very important pain relief with a reduction of the majority of allodynic territory, which resulted in temporarily increased quality of life and reduction of symptoms associated with depression, arising from the chronic pain condition. After three consecutive procedures, there was a very significant reduction of the allodynia area (Figure 1B), allowing for greater ROM and functionality of the upper limb, despite signs and symptoms relapsing around two to three months after the last intervention. No adverse effects were recorded, namely respiratory repercussions, due to inadvertent phrenic nerve block.

Given the positive outcome, a peripheral nerve stimulation system was implanted to produce peripheral neuromodulation and achieve enhanced long-term pain management, with the patient controlling the device, in a home setting.

## Discussion

SCP iatrogenic injury can result from dissection and surgical exploration of the neck region, potentially leading to post-surgical chronic pain. Neuropathic symptoms are most often experienced in the distribution of dermatomes corresponding to the cervical plexus superficial nerves [10]. The prevalence of this type of pain after various surgeries in the cervical region is quite variable and depends on the performed procedure [5].

The SCP is essential for sensory innervation of the cervical region, and it should be accessed by physical examination since it can be addressed with pain interventional techniques [11]. The use of ultrasound in the head and neck region has been expanding, allowing easy identification of several important landmarks, including muscles, cervical vertebrae, vessels, nerves, and cervical fascia. It also allows visualization of post-surgical changes such as neuromas, and fibrosis adhesion phenomena, and it allows guidance for different therapies such as nerve blocks, hydrodissection, and application of other treatment modalities such as pulsed radiofrequency and cryoneurolysis [12,13].

The main complications of cervical plexus nerve block are the inadvertent injection of local anesthetic into deeper structures, including the phrenic nerve, brachial plexus, or recurrent laryngeal nerve [14]. Although there is no precise estimation of complication rates, several authors report that these are easily avoided with standard precautions, the most important being the injection of local anesthetic only when there is direct visualization of the needle tip [15,16].

Nerve blocks have been shown effective in short-term relief of neuropathic pain and they can be repeated until the pain improves significantly. If not, other interventions (neurolysis, spinal cord stimulation, peripheral nerve stimulation, pulsed radiofrequency injury, or radiofrequency thermocoagulation) are viable alternatives [14].

This case report highlights the importance and added value of using SCP block in a patient with territorial neuropathic pain, refractory to conventional treatment. Topographic correlation of cutaneous nerve supply and distribution of pain provided a basis for diagnosis and to perform the plexus block. These procedures contributed to pain relief for a few weeks, and potential benefits beyond this period can possibly be explained by the breaking of the pain cycle, in addition to the use of steroids, as an adjuvant.

## Conclusions

Given the SCP injury, suspected due to surgery/extrinsic compression phenomena of its sensory branches due to the scarring process, a new approach of ultrasound-guided hydrodissection of the small peripheral sensory branches (set of hypoechoic nodules) located superficially to the prevertebral fascia and deeply to the lateral edge of sternocleidomastoid muscle was performed. This approach allowed temporary pain relief in an extensive cutaneous area from the cervical region to the shoulder/pectoral region. Simple SCP block is commonly used in locoregional anesthesia (thyroid and carotid surgery) but, as described, it can also be used in interventional pain medicine for treating neuropathic pain referred to the anterior part of the neck, face, or mandible, specifically on those patients who have undergone cervical surgery.
